# Psychometric properties of a new treatment expectation scale in rheumatoid arthritis: an application of item response theory

**DOI:** 10.1186/s12891-015-0690-3

**Published:** 2015-09-04

**Authors:** Fowzia Ibrahim, Salma Ayis, Darija Hofmann, Diana Rose, Til Wykes, Andrew Cope, David L. Scott, Heidi Lempp

**Affiliations:** Academic Department of Rheumatology, Faculty of Life Sciences and Medicine, King’s College London, London, UK; Department of Primary Care and Public Health Sciences, Faculty of Life Sciences and Medicine, King’s College London, London, UK; Department of Health Services & Population Research, Institute of Psychiatry, Psychology and Neuroscience, King’s College London, London, UK; Department of Psychology, Institute of Psychiatry, Psychology and Neuroscience, King’s College London, London, UK; Academic Department of Rheumatology, King’s College London, Weston Education Centre, 10, Cutcombe Road, Denmark Hill, London, SE5 9RJ UK

## Abstract

**Background:**

Patient-generated health outcome measures are important in the assessment of long-term treatment goals for Rheumatoid Arthritis (RA), but few psychometrically sound measures are available. The MAPLe-RA (**M**easuring **A**ctual **P**atient-**L**ed **e**xpectations in RA) is a new questionnaire and its psychometric properties are not investigated. This study aims to examine these properties for each of the items using Item Response Theory (IRT) .

**Methods:**

Participants were included if they completed the scale (MAPLe-RA). A one parameter (Rasch) model and a two parameter logistic (2PL) model were applied to these data using M-plus software.

**Results:**

One hundred thirty-eight patients with RA were included in the analysis. MAPLe-RA scale comprised of 21 items, the mean score was 71 (20.28) ranging from 0 to 105. Most items operated in the high expectations part of the items characteristics curves (ICC). Item discrimination varied widely, items with the highest discrimination capacity from the three domains were: pain (physical domain); control of my RA (self-management) and maintaining social role (psycho-social domain); feeling better overall and involvement in treatment decision making (impact of new treatment domain).

**Conclusion:**

RA patients’ expectations of treatment are higher in the physical and psycho-social domains and less so in the impact of new treatment domain.

**Electronic supplementary material:**

The online version of this article (doi:10.1186/s12891-015-0690-3) contains supplementary material, which is available to authorized users.

## Background

Patient- generated health outcome measures play an important role in the assessment of long-term treatment goals, for people experiencing Rheumatoid arthritis (RA) and are therefore well positioned to be utilised in assessing new treatments. In RA, there is no gold standard measure for the assessment of patient’s expectations at time of diagnosis or before commencing treatment. To fill this gap we developed a new patient-generated expectancy measure, called **M**easuring **A**ctual **P**atient-**L**ed **e**xpectations in RA (MAPLe-RA) scale [1]. Item response theory (IRT) is an approach that emphasizes the influence of the individual’s qualities as well as the items qualities, in a test, or in a questionnaire. The method was originated in education where individual qualities may reflect abilities, was then extended to other applications, with well-known examples in medicine and psychology [2–4]. In this study the underlying construct is patients’ expectations, and the method was used to understand the psychometric properties of the individual items [5, 6]. Although IRT method has been applied in several long-term conditions to assess the properties of outcome measures and questionnaires [7], it is rarely used in patient-generated measures in RA [8, 9]. For the development of new scales, traditionally, a factor extraction method based on Eigenvalues is used [10, 11] to explore the number of domains, and the strength of association of items within domains. IRT in addition provides important details, on psychometric properties of each item, including, the difficulty and the discrimination of these properties. MAPLe-RA is a new questionnaire and its psychometric properties have not yet been investigated. This study aims to examine these properties for each of the items using IRT.

## Methods

The development stages of MAPLe-RA were published elsewhere [1]. In brief, stage one of the study: three repeated focus groups and two expert panels with RA patients were conducted by a patient researcher. Stage two: a feasibility study of the draft scale with 22 consecutive outpatient attendees over 1 week was conducted and stage three was the psychometric analysis, and that the results are presented here. MAPLe-RA scale, comprised of 21 items, and the response options were given in a Likert scale from 5 to 0. High scores refer to better treatment expectations. The scale is intended to measure expectations of treatment in three domains: physical, psychosocial and impact of treatment. Table 1 shows how the MAPLe-RA questionnaire is scored. MAPLe-RA was approved by the National Research Ethics Committee London-Central (REC reference number 10/HO718/82). All participants provided informed consent.

**Table 1:** MAPLe-RA questionnaire

A. The Physical domain (physical impact of RA): with the new treatment, I expect:						
Q1: The swelling of the joints to be	Much better	Better	Same	Worse	Much Worse	non-applicable
Q2: The pain to be	Much better	Better	Same	Worse	Much Worse	non-applicable
Q3: My morning stiffness to be	Much better	Better	Same	Worse	Much Worse	non-applicable
Q4: My mobility to be	Much better	Better	Same	Worse	Much Worse	non-applicable
Q5: My fatigue to be	Much better	Better	Same	Worse	Much Worse	non-applicable
Q6: The visible signs of RA (e.g. deformities on my hands) to be	Much better	Better	Same	Worse	Much Worse	non-applicable
Q7: The joint damage to be caused by RA	Much better	Better	Same	Worse	Much Worse	non-applicable
Questions for physical domain are coded from 5 = Much better to 0 = non-applicable; overall score for the physical domain ranges between 0 and 35.						
B. The Psycho-social domain (emotional wellbeing and social aspects of RA): with the new treatment, I expect:						
Q1: To be able to maintain my independence (e.g. not needing to ask for help making tea)	Much more than usual	More than usual	Same	Worse than usual	Much worse than usual	non-applicable
Q2: Improvements in my general health in order for me to be able to go back to work and/ or stay in salaried employment: (Please tick here if not applicable)	Much more than usual	More than usual	Same	Worse than usual	Much worse than usual	non-applicable
Q3: My everyday activities (e.g. shopping) to be facilitated:	Much more than usual	More than usual	Same	Worse than usual	Much worse than usual	non-applicable
Q4: To feel in control of my RA self manage (e.g. diet)/ cope (e.g. frustration) alongside medical treatment	Much more than usual	More than usual	Same	Worse than usual	Much worse than usual	non-applicable
Q5: To be able to maintain my social roles (e.g. supporting family/going out with friends)	Much more than usual	More than usual	Same	Worse than usual	Much worse than usual	non-applicable
Q6: My emotional wellbeing (e.g. mood) to be	Much more than usual	More than usual	Same	Worse than usual	Much worse than usual	non-applicable
Questions for the Psycho-social domain are coded from 5 = Much more than usual to 0 = non-applicable; overall score for the psycho-social domain range between 0 and 30.						
C. Impact of new Treatment (Overall Expectations on Impact of Treatment (care delivery)): with the new treatment I expect it:						
Q1: To make me feel better overall despite side effects	Strongly Agree	Agree	Neither Agree nor Disagree	Disagree	Strongly Disagree	non-applicable
Q2: To reduce the likelihood of surgery	Strongly Agree	Agree	Neither Agree nor Disagree	Disagree	Strongly Disagree	non-applicable
Q3: To prevent other physical complications	Strongly Agree	Agree	Neither Agree nor Disagree	Disagree	Strongly Disagree	non-applicable
Q4: To come with detailed information from the medical staff:	Strongly Agree	Agree	Neither Agree nor Disagree	Disagree	Strongly Disagree	non-applicable
Q5: To allow me to be involved in the treatment decision making with the clinical staff	Strongly Agree	Agree	Neither Agree nor Disagree	Disagree	Strongly Disagree	non-applicable
Q6: To include regular physical (e.g. hands and feet) assessments	Strongly Agree	Agree	Neither Agree nor Disagree	Disagree	Strongly Disagree	non-applicable
Q7: To include regular emotional wellbeing assessments	Strongly Agree	Agree	Neither Agree nor Disagree	Disagree	Strongly Disagree	non-applicable
Q8: To allow me to not have to change medication so often	Strongly Agree	Agree	Neither Agree nor Disagree	Disagree	Strongly Disagree	non-applicable
Questions for the impact of new treatment are coded from 5 = strongly agree to 0 = non-applicable; overall score for the impact of new treatment scores range between 0 and 40.						

Computing and interpreting the MAPLe-RA score

There are two steps in computing the overall score of MAPLe-RA

1. Sum the scores for each domain

2. Sum the scores for all the domains. This yields a MAPLe-RA score ranging between 0 and 105. The highest score representing high expectations of new treatment and vice versa

Non-applicable response category refers to patients who are not newly diagnosed with RA or not changing treatment

### Data analysis

The demographic information of participants are described in Table 2, using proportions, means and standard deviations (SD) as appropriate. The item responses for all the 21 items were skewed towards ‘better’ and ‘much better’. For the factor analysis we used the original scales of the items. However, for the purpose of the IRT model, we dichotomised the items by collapsing the (i) ‘better’ and ‘much better’ responses together and coded as 1; and (ii) ‘worst’, ‘much worst’, ‘same’ and ‘non-applicable’ responses coded as 0. We fitted a one parameter (Rasch) model which, assumes that the items are equally discriminating but with varying difficulty, and a two parameter logistic (2PL) model that assumes the items have a varying ability to discriminate among patients with different levels of the underlying construct [13, 14]. Uni-dimensionality was assumed as a priori and was further assessed using maximum likelihood method as well as principal-component factor methods. We present the results obtained from the 2PL model, as these provide more desired information, including items’ difficulty and discrimination [15, 16]. The model fits the data well as assessed by the Akaike information criterion (AIC) and Bayesian information criterion (BIC). We employed the Item-characteristic Curve (ICC) to evaluate the profile of each item within the scale and to assess the relationship between the predicted patients’ response to an individual item and the underlying construct (expectations). For all the analyses we used M-Plus statistical software.

**Table 2:** Demographic information of participants in the study

	*n* = 138
	n (%)
Mean age (SD)	54 (14.30)
Gender	
Female	101 (73 %)
Male	37 (27 %)
Ethnicity	
White	73 (53 %)
Other	65 (47 %)
Disability	
Yes	48 (35 %)
No/Unsure	90 (65 %)

*SD* standard deviation

The two-parameter logistic model suits binary responses and may be described as:

1 $$ p\left({x}_j=1\left|\theta \right.\right)=\frac{1}{1+ \exp \left\{-{a}_j\left(\theta -{\beta}_j\right)\right\}} $$

Where *x*_*j*_ is the observed response to item *j*, *α*_*j*_ is the slope parameter, *β*_*j*_ is the difficulty (location) of item j, and θ is the underlying construct being measured (expectations).

## Results

### Study participants

A total of 160 outpatient attendees were invited to take part in phase 3 of the study, 138 (86 %) consented and completed the MAPLe-RA questionnaire. The mean age was 54 (SD = 14.30) years; 101 (74 %) were women, 73 (53 %) were of white ethnicity and 48 (38 %) reported being registered disabled (Table 2).

### MAPLe-RA scale properties

In stage one and two of the scale development, patients identified 21 dimensions of new treatment expectations, grouped into (i) physical (ii) psycho-social and (iii) expectations relating to impact of treatment. This resulted in a draft questionnaire assessed in the feasibility study and subsequent stage three analysis.

The overall mean score of MAPLe-RA 21 items, was 71 (SD: 20.28; range 0 to 105). The means for the 3 domains, separately were: physical (7 items), mean 24.40 (SD: 7.21), psycho-social (6 items), mean 17.51 (SD: 8.17) and impact of new treatment domain (8 items) mean 30.76 (SD: 7.02).

Exploratory factor analysis identified that all items had strong positive associations with the first factor, weak associations in most items with the second factor, and negative associations with items 13–21 (Additional file 1: Table S1). The Eigen value for the first factor was 6.25, proportion of variance explained was 77 %, supporting the uni-dimensionality of the items.

Most items had high rates of “yes” or positive responses, in IRT context, these have low difficulty parameters, most patients would pick, and these seem to describe the majority of patients’ expectations. Item discrimination on the other hand reflects the strength of the association of an item with the underlying construct, items with high discrimination are better at differentiating respondents at the location point; small changes in the underlying construct (expectation) leads to large changes in the probability of endorsing the item (response = yes), and vice versa for items with low discrimination. These responses varied widely, and the most powerful two items from the three domains were: “swelling of the joints” and “pain” in the physical domains, discriminations: 1.38 (0.58) and 1.74 (0.82).

In the psycho-social domains, **“**to maintain social role” and “emotional wellbeing” were the two items with highest discrimination: 1.87 (0.63) and 1.77 (0.51), respectively. The two items with the highest discrimination in impact of new treatment domain were “feeling better overall” and “involvement in treatment decision making”, 1.47 (0.38) and 1.41 (0.52) respectively, and other items had lower discrimination. Details for all items are presented in (Table 3). Within the physical domains, two items had a difficulty that was not different from zero, namely “visible signs of RA” and “joint damage”, and the two items also had very low discrimination. The model fit was high in both the BIC (2807.48) and AIC (2743.08), which indicates a good fit of the two-parameter logistic model.

**Table 3:** Item characteristics (difficulties and discrimination) for MAPLe-RA scale

	Item discriminations		Item difficulties	
Items	Estimate (SE)	P-value	Estimate (SE)	P-value
Physical domain				
Swelling of the joints	1.38 (0.58)	0.018	−1.22 (0.30)	<0.001
Pain	1.74 (0.82)	0.034	−1.25 (0.30)	<0.001
Morning stiffness	1.25 (0.47)	0.008	−1.00 (0.25)	<0.001
Mobility	1.04 (0.41)	0.012	−1.16 (0.31)	<0.001
Fatigue	0.87 (0.26)	0.001	−0.96 (0.25)	<0.001
Visible signs of RA	0.49 (0.16)	0.002	0.19 (0.24)	0.427
Joint damage	0.40 (0.15)	0.006	−0.62 (0.34)	0.066
Psycho-social domain				
Maintain my independence	1.46 (0.39)	<0.001	−0.14 (0.13)	0.270
Improvements in my general health	0.85 (0.23)	<0.001	0.06 (0.17)	0.712
Everyday activities	1.27 (0.40)	0.001	−0.32 (0.14)	0.027
To feel in control of my RA self-manage	1.64 (0.49)	0.001	−0.46 (0.13)	0.001
To maintain my social roles	1.87 (0.63)	0.003	−0.30 (0.12)	0.016
My emotional well-being	1.77 (0.51)	0.001	−0.42 (0.13)	0.001
Impact of new Treatment				
Feel better overall	1.47 (0.38)	<0.001	−1.02 (0.20)	<0.001
Reduce the likelihood of surgery	0.89 (0.27)	0.001	−1.19 (0.29)	<0.001
To prevent other physical complications	0.70 (0.27)	0.008	−1.47 (0.44)	0.001
To come with detailed information from the medical staff	1.16 (0.45)	0.010	−1.36 (0.34)	<0.001
Decision making with the clinical staff	0.87 (0.34)	0.010	−1.65 (0.47)	<0.001
Regular physical assessment	1.41 (0.52)	0.007	−1.36 (0.29)	<0.001
Regular emotional well-being	0.81 (0.26)	0.001	−0.90 (0.25)	<0.001
Not to have to change medication	0.52 (0.18)	0.005	−1.14 (0.40)	0.004

p-value is for the two parameter model

*SE* standard error

**Fig. 1:**
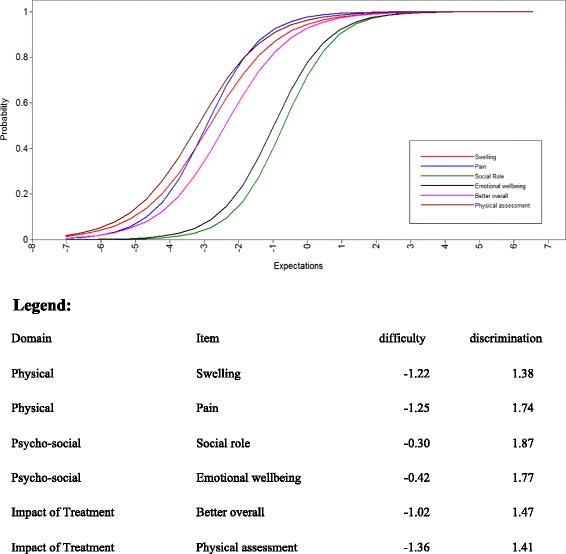
Two Parameter Logistic Model (2PLM) item characteristics ccurves (ICC), for two items from each domain of the MAPLe-RA scale

Figure 1 shows the Item Characteristic Curves (ICC) that represents the respondents’ expectations (underlying construct) in relation to the probability of endorsing an item and is presented graphically for two items with the highest discrimination from each domain.

## Discussion

MAPLe-RA is a new questionnaire that was not yet validated and its items were not examined. In this study, we used factor analysis to assess the uni-dimentionality and IRT to describe the properties of the items. The study has shown that RA patients have high expectations from their treatment. These are particularly high in the physical and psycho-social domains rather than in the impact of new treatment domain. For the latter, most items were unlikely to be endorsed by patients with less than average expectations in this RA study cohort.

### Strength and limitations of the study

All domains have shown items with strong association with the underlying construct (expectation), two to three items from each domain, may be considered as good candidates that differentiate between patients’ responses. Several items however, appeared to be redundant (e.g. visible signs of RA; not to have to change medication), as they did not show strong association with the underlying construct.

The IRT method is superior to the traditionally factor extraction methods based on Eigenvalues [10, 11]. It is also a suitable way to employ when an instrument includes response categories that have several levels. In this study, the method determines whether the categories perform as they were envisioned and/or whether to collapse the responses into fewer categories [17]. The advantage of using IRT is that of an underlying construct, that gives items different weights, depending on the response pattern and the frequency of response to each item, and values instead of sum scores [12, 18]. This technique has been successfully applied in the development or the evaluation of new measures in patient-reported outcomes [19, 20]. To our knowledge the IRT method has not been applied in many Rheumatology related scale studies [21].

While the results of the new instrument appeared to have a very good reliability, it is important to interpret the findings with caution. This analysis was an exploratory phase of the scale development stages. The sample size was under powered for IRT 2PL model and the population studied was homogeneous from one RA clinic only. Although, there were some redundant items, we chose to keep these in the analysis to avoid making inappropriate decisions and or conclusions at this early stage.

Other studies have found similar results in that new measure of patient’ expectations in general need validation in larger multi-centre studies [22]. We acknowledge that further analysis is necessary, thus MAPLe-RA is currently included in a national longitudinal observational study of patients with early Rheumatoid Arthritis with a diversity of socio-demographic characteristics and a long term follow up of 18 months, to be completed in 2015. This large multi-centre study will allow us to conduct a confirmatory analysis of the new measure as well as to assess if patients expectations change over time.

## Conclusions

This study extends the evidence on the value of IRT models in the assessment of health outcomes and patient-generated measures. The result highlights that RA patients’ treatment expectations are higher in the physical and psycho-social domains and less so in the impact of new treatment domain. RA patients expect high degree of involvement in their care from health care providers, and that they rate highly, controlling their pain and emotional well-being.

## Additional file

Additional file 1: Table S1. Factor loading for MAPLe-RA scale. (DOCX 17 kb)
