# Supplementary material for: Psychometric properties of a new treatment expectation scale in rheumatoid arthritis: an application of item response theory
Source: BMC Musculoskelet Disord. 2015 Sep 4;16:239. doi: 10.1186/s12891-015-0690-3 (PMC4559926; doi:10.1186/s12891-015-0690-3)
Supplement: Additional file 1: Table S1. — Factor loading for MAPLe-RA scale. (DOCX 17 kb) [file 12891_2015_690_MOESM1_ESM.docx]

**Additional file 1: Table S1.** Factor loading for MAPLe-RA scale.

| Items | Factor1 | Factor2 |
| --- | --- | --- |
| **Physical domain** |  |  |
| Swelling of the joints | 0.6388 | 0.3891 |
| Pain | 0.585 | 0.4215 |
| Morning stiffness | 0.5387 | 0.4795 |
| Mobility | 0.4349 | 0.5213 |
| Fatigue | 0.4304 | 0.1763 |
| Visible signs of RA | 0.2703 | 0.3634 |
| Joint damage | 0.2683 | 0.3316 |
| **Psycho-social domain** |  |  |
| Maintain my independence | 0.5294 | 0.0589 |
| Improvements in my general health | 0.3645 | 0.1030 |
| Everyday activities | 0.4848 | 0.1669 |
| To feel in control of my RA self-manage | 0.6239 | 0.1799 |
| To maintain my social roles | 0.5648 | 0.0722 |
| My emotional well-being | 0.5809 | -0.0206 |
| **Impact of new Treatment** |  |  |
| Feel better overall | 0.6461 | -0.1910 |
| Reduce the likelihood of surgery | 0.5164 | -0.1135 |
| To prevent other physical complications | 0.5782 | -0.3475 |
| To come with detailed information from the medical staff | 0.6912 | -0.3797 |
| Decision making with the clinical staff | 0.6396 | -0.4785 |
| Regular physical assessment | 0.6822 | -0.0958 |
| Regular emotional well-being | 0.5617 | -0.2925 |
| Not to have to change medication | 0.5530 | -0.2165 |
